# Porcine model elucidates function of p53 isoform in carcinogenesis and reveals novel *circTP53* RNA

**DOI:** 10.1038/s41388-021-01686-9

**Published:** 2021-02-18

**Authors:** Guanglin Niu, Isabel Hellmuth, Tatiana Flisikowska, Hubert Pausch, Beate Rieblinger, Alexander Carrapeiro, Benjamin Schade, Brigitte Böhm, Eva Kappe, Konrad Fischer, Bernhard Klinger, Katja Steiger, Reiner Burgkart, Jean-Christophe Bourdon, Dieter Saur, Alexander Kind, Angelika Schnieke, Krzysztof Flisikowski

**Affiliations:** 1 Chair of Livestock Biotechnology, Technische Universität München, Munich, Germany; 2 Animal Genomics, ETH Zurich, Zurich, Switzerland; 3 Department of Pathology, Bavarian Animal Health Service, Poing, Germany; 4 School of Medicine, Institute of Pathology, Technische Universität München, Munich, Germany; 5 Klinik und Poliklinik für Orthopädie und Sportorthopädie, Klinikum rechts der Isar, Technische Universität München, Munich, Germany; 6 Jacqui Wood Cancer Centre, School of Medicine, University of Dundee, Dundee, UK; 7 Department of Internal Medicine II, Klinikum rechts der Isar, Technische Universität München, Munich, Germany

**Keywords:** Bone cancer, Gene regulation

## Abstract

Recent years have seen an increasing number of genetically engineered pig models of human diseases including cancer. We previously generated pigs with a modified *TP53* allele that carries a Cre-removable transcriptional stop signal in intron 1, and an oncogenic mutation *TP53*^*R167H*^ (orthologous to human *TP53*^*R175H*^) in exon 5. Pigs with the unrecombined mutant allele (fl*TP53*^*R167H*^) develop mainly osteosarcoma but also nephroblastomas and lymphomas. This observation suggested that *TP53* gene dysfunction is itself the key initiator of bone tumorigenesis, but raises the question which aspects of the *TP53* regulation lead to the development of such a narrow tumour spectrum. Molecular analysis of p53 revealed the presence of two internal *TP53* promoters (Pint and P2) equivalent to those found in human. Consequently, both pig and human express *TP53* isoforms. Data presented here strongly suggest that P2-driven expression of the mutant R167H-Δ152p53 isoform (equivalent to the human R175H-Δ160p53 isoform) and its circular counterpart *circTP53* determine the tumour spectrum and play a critical role in the malignant transformation in fl*TP53*^*R167H*^ pigs. The detection of Δ152p53 isoform mRNA in serum is indicative of tumorigenesis. Furthermore, we showed a tissue-specific p53-dependent deregulation of the p63 and p73 isoforms in these tumours. This study highlights important species-specific differences in the transcriptional regulation of *TP53*. Considering the similarities of *TP53* regulation between pig and human, these observations provide useful pointers for further investigation into isoform function including the novel *circTP53* in both the pig model and human patients.

## Introduction

In human, osteosarcomas (OS) is the major form of primary bone cancer [1]. It predominantly affects young people and is highly malignant, requiring aggressive surgical resection and cytotoxic chemotherapy [2]. The 5-year survival rate has remained unchanged over the past 20 years, at ~60% for patients with primary osteosarcoma and ~20% for patients with metastatic disease [3]. Most OS are sporadic and of unknown cause, but increased incidence is associated with Li–Fraumeni syndrome caused by germ line mutation of *TP53* [4, 5].

The role of *TP53* mutations in numerous cancers has been extensively documented [6]. Yet, potentially important aspects of *TP53* gene function still remain unclear, including the role of various p53 isoforms. Human *TP53* is known to express at least 9 different mRNA transcripts [7] and at least 12 protein isoforms [8], with transcription initiated by two promoters: P1 at the 5’ end and P2 in intron 4; alternative splicing across introns 2 and 9; and alternative translation initiated at internal start codons 40, 133 and 160. The internal promoter P2 originates the Δ133p53 and Δ160p53 isoforms in humans. While the function of Δ160p53 is less studied, the Δ133p53 is involved in the regulation of replicative cellular senescence [9], angiogenesis, cytokine secretion/immune response and tumour progression in some cancer types [10].

Pigs have become an important animal model for human cancers research, translational studies and preclinical trials [11, 12]. We have generated genetically engineered (GE) pigs with mutations in key tumour suppressor genes and proto-oncogenes [13] to facilitate preclinical studies [14]. As part of this programme we generated pigs modelled on GE mice carrying a Cre-inducible oncogenic mutant *Trp53*^*R172H*^ allele, in which a floxed transcriptional stop cassette is inserted into *Trp53* intron 1 to block transcription of the mutated allele [15]. The latent allele can be experimentally activated by Cre recombinase to express mutant p53^R172H^ and model spontaneous somatic *Trp53* mutation in chosen organs. These mice have been used successfully to model a series of cancers, e.g., pancreatic, breast and lung [16–19]. Similarly, our pig line carries an engineered endogenous *TP53* gene with a floxed transcriptional stop signal in intron 1 and a point mutation resulting in an arginine to histidine substitution at codon 167 in exon 5 (*TP53*^*R167H*^, orthologous to mouse *Trp53*^*R172H*^ and human *TP53*^*R175H*^) [20]. As this pig line was being established it became apparent that animals with the latent non-induced allele (designated here as fl*TP53*^*R167H*^) in both heterozygous and homozygous form developed OS [21] and less frequently kidney tumours and lymphomas.

This phenotype contrasts sharply with that reported for mice carrying the apparently equivalent non-induced *Trp53*^*R172H*^ allele, which mostly develop lymphoma [22]. None of the murine *Trp53* mutant strains so far generated show a preponderance of OS in the tumour spectrum [22, 23]. These include Cre-inducible point mutations [23], partial deletions of the *Trp53* coding region [15] and transgenic lines that overexpress mutant *Trp53*^*R270H*^ and *Trp53*^*R172H*^ from exogenous promoters [24]. To increase the incidence of OS, several mouse strains expressing osteoblast- and mesenchymal-specific Cre coupled with conditional *p53* and *Rb1* mutant alleles were generated [25].

These findings indicate a species-specific difference in p53 gene regulation. Differences in the p53 isoforms and internal promoters have been described for humans, primates, zebrafish, drosophila and mouse [8]. Here we show that unlike the mouse, the porcine *TP53* locus contains internal Pint and P2 promoters equivalent to those found in human, resulting in expression of Δ152p53α isoform and its circular counterpart *circTP53*. Both are involved in the development of OS and others tumours in the fl*TP53*^*R167H*^ pigs and the detection of Δ152p53α isoform in serum is indicative of tumorigenesis. A tissue-specific p53-dependent deregulation of p63 and p73 isoforms could be observed in these tumours.

## Results

### Cancer spectrum in flTP53^R167H^ pigs

Since our publication describing the generation of fl*TP53*^*R167H*^ pigs [20], a total of 29 fl*TP53*^*R167H/+*^ heterozygous and 10 fl*TP53*^*R167H/R167H*^ homozygous pigs were examined by necropsy. All had reached sexual maturity. Animals were sacrificed as soon as they showed symptoms effecting their wellbeing or in case of the heterozygous animals latest at the age of 36 months. By this time 18 of the 29 heterozygous animals had developed tumours, all of which were classified histologically as osteoblastic OS. Spontaneous OS is very rare in wild-type pigs [21, 26]. All homozygous pigs had OS by the age of 16 months or earlier and five of these (aged 7–14 months) also had nephroblastoma (kidney) and diffuse large B-cell lymphomas (spleen). In each case the anatomical location and histological analyses of the tumours resembled those of the human juvenile cancer.

### Identification of a Δ152p53α isoform in pigs

The observation that the tumour repertoire was restricted to a few tissue types (100% OS, 22% nephroblastoma and 17% B-cell lymphomas) leads to the question which aspects of *TP53* expression or absence thereof support the formation of mainly OS rather than other tumour entities? To answer this question, we first establish if expression of the floxed fl*TP53*^*R167H*^ allele was silenced in all organs.

**Fig. 1:**
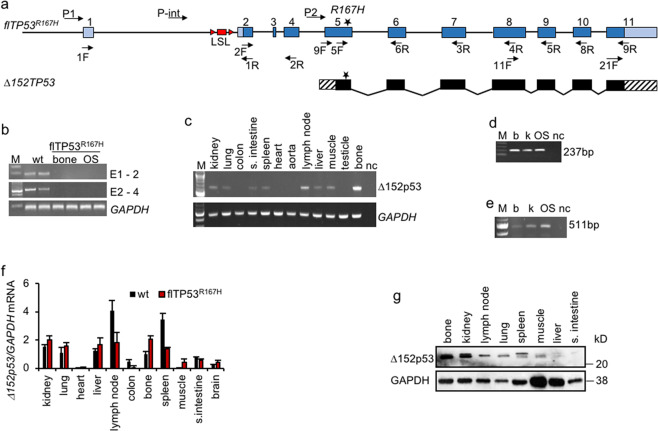
Identification of Δ152p53α isoform in pigs. **a** Schematic representation of the *TP53* gene and the Δ152p53α mRNA. **b** Expression analysis across exon 1 to 2 (186 bp), exon 2 to 4 (591 bp) of bone samples from wild-type pigs and healthy bone and OS samples from *flTP53*^*R167H/R167H*^ pigs showing lack of expression for mutant TP53. **c** Expression analysis across exon 5 to 11 (492 bp) for 11 different organ samples from *flTP53*^*R167H/R167H*^ pigs. **d** 5’ RACE using a reverse primer hybridising to exon 6 of *TP53* and resulting in a 237 bp fragment and **e** 3’ RACE using forward primer hybridising to exon 11 of *TP53* resulting in a 511 bp fragment. Healthy bone (b), kidney (k) and osteosarcoma (OS) samples from *flTP53*^*R167H/R167H*^ pigs. M marker, nc negative control. **f** Quantitative RT-PCR analysis using primers hybridising to extended exon 5 and exon 6 and detecting the Δ152p53 isoform mRNA expression in different tissues of *flTP5*^*R167H/R167H*^ (*n* = 6) and wild-type (*n* = 3) pigs*. GAPDH* was used as a reference gene to calculate the relative expression levels. **g** Detection of Δ152p53 isoform by western blot analysis using SAPU antibody in different tissues from *flTP5*^*R167H/R167H*^ pigs.

A series of RT-PCR primers hybridising to different porcine *TP53* exons (Fig. 1a) were used to screen RNA samples from various organs (*n* = 11) of fl*TP53*^*R167H/R167H*^ homozygous pigs (*n* = 3). No mRNA expression was detected using RT-PCR primers specific for exon 1 to exon 2 (1F/1R), or exon 2 to 4 (2F/2R) in all tissues analysed including bone and OS samples (Fig. 1b), confirming that the LSL efficiently blocked transcription. However, RT-PCR using primers between exons 5 and 11 (5F/9R, see Fig. 1a) revealed a tissue-specific expression (Fig. 1c), where the strongest RT-PCR signal was obtained for bone, lymph node and kidney tissues. This indicated the presence of a promoter element in intron 4 similar to human. No expression of the short *TP53* mRNA was detected in heart and aorta. To determine the start and termination of the porcine *TP53* isoform 5’ and 3’ rapid amplification of cDNA end (RACE) analysis was performed (Fig. 1d, e). This placed the start of the isoform 11 base pairs upstream of exon 5 with a possible ATG at amino acid position 152 (Δ152p53 isoform), which translates in frame and terminates at the same stop codon as the full length p53 and corresponds to *TP53α* isoform (Supplementary Fig. 1). The identified Δ152p53α mRNA has a length of 1295 bp; the nucleotide sequence was verified by sequencing.

### Expression of Δ152p53α isoform is highest in organs susceptible to cancer development

By using primers (9F/6R) specific for the Δ152p53α isoform its expression was quantified by qPCR in different healthy tissues (*n* = 11) from fl*TP53*^*R167H*^ homozygous (*n* = 10) and wild-type (*n* = 3) pigs. The highest Δ152p53α mRNA expression in fl*TP53*^*R167H*^ homozygous pigs was observed in lymph nodes, spleen, kidney and bone (Fig. 1f), all organs prone to tumour development in mutant pigs. Except for lymph node, colon and small intestine the Δ152p53α mRNA expression was generally lower in wild-type samples, than in fl*TP53*^*R167H*^ homozygous samples.

### The porcine Δ152p53α isoform encodes a 30kDa protein

The methionine codon 152 in pigs corresponds to codon 160 in human p53, which is located within a highly conserved Kozak sequence [27]. An in silico analysis predicted that the 1295 bp mRNA encodes a 224 amino acid N-terminal-truncated Δ152p53α isoform using the same reading frame as full length p53. The predicted porcine isoform shares 88% amino acid sequence homology with the human Δ160p53α isoform, which uses the Δ133/160p53 alternative transcriptional initiation site in exon 5 [27] and contains part of the highly conserved p53 DNA binding region [8]. To determine whether the porcine *TP53* alternative transcript can produce Δ152p53α protein, western blot analysis of different healthy tissue samples from fl*TP53*^*R167H/R167H*^ homozygous pigs was performed using the polyclonal sheep SAPU antibody recognising all human p53 isoforms. Two bands (doublet) of ~30 kD were detected (Fig. 1g). As with the mRNA highest protein expression was observed in bone, kidney, lymph node and spleen. The size of the western blot bands was comparable to the human Δ160p53 isoforms [27], and most likely represent splice variant or post-translational modifications of R167H-Δ152p53 proteins.

### Identification of internal promoters in the porcine *TP53*

Results above clearly indicated the presence of at least one internal promoter. To determine the location of all possible internal promoters, nucleotide sequence alignment was carried out between the porcine (NC_010454, GenBank Sscrofa 11.1) and human (NC_000017, Genbank GRCh38.p12) *TP53* gene. Apart of exons, three genomic regions with high homology (>70% similarity) were detected (–1877 to +11 bp, +2125 to +3200 bp and +9985 to +10,470 bp; relative to the major transcription start site). These porcine *TP53* regions correspond to the locations of the P1 (5’end), Pint (intron 1) and P2 (intron 4) promoters in human *TP53*. As for the human sequence, the porcine intron 4 sequence (P2) contains binding sites for the transcription factors NFIC, Hltf and SPI1. In contrast, the sequence of mouse *Trp53* intron 4 showed less than 50% homology to human *TP53*, and lacks binding sites for these transcription factors (Supplementary Fig. 2). Interestingly, the NFIC transcription factor regulates osteoblast differentiation [28], and can promote or suppress the development of various cancers [29] via epigenetic changes [30]. The SPI1 transcription factor regulates alternative transcription of target genes, and both Hltf and SPI1 are frequently mutated in paediatric cancers [31, 32].

**Fig. 2:**
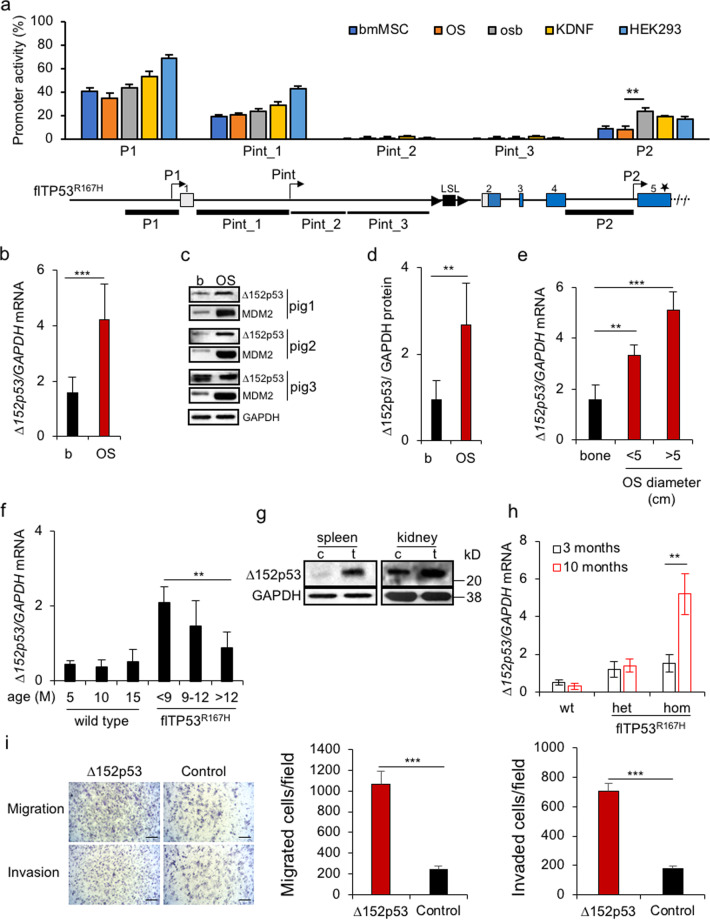
Identification of porcine *TP53* promoters and expression of Δ152p53α isoform in osteosarcomas. **a** Dual-luciferase assay: luciferase was expressed from the SV40 promoter and used to normalise the Renilla expression under the control of the putative promoter fragments. Their location relative to the gene structure is depicted. Values represent mean ± standard deviation, six transfections per construct. Promoterless luciferase vector was used as a negative control. bmMSC bone marrow mesenchymal stem cells, osb porcine fl*TP53*^*R167H/R167H*^ osteoblasts, OS porcine *flTP53*^*R167H/R167H*^ osteosarcoma cells, KDNF porcine kidney fibroblasts, HEK293 human embryonic kidney cell line. **b** Quantitative PCR results of Δ152p53α mRNA expression in OS (*n* = 48) and matched healthy bone samples. **c** Representative western blots showing Δ152p53α protein and MDM2 expression in OS and healthy matched bone samples of homozygous *flTP53*^*R167H*^ pigs. **d** Quantitative measurements of proteins in OS (*n* = 10) and healthy matched bone samples from homozygous *flTP53*^*R167H*^ pigs. **e** The Δ152p53α mRNA expression in small (<5 cm, *n* = 29) vs. large (>5 cm, *n* = 19) tumours. **f** Age-dependent Δ152p53α mRNA expression in healthy bones of homozygous fl*TP53*^*R167H*^ (*n* = 10) and wild-type (*n* = 3) pigs. **g** Western blots showing Δ152p53α isoform expression kidney and spleen tumours and healthy matched tissues of *flTP5*^*R167H/R167H*^ pigs. **h** QPCR analysis of Δ152p53α mRNA expression in blood exosomes from fl*TP53*^*R167H*^ heterozygous (*n* = 6), homozygous (*n* = 6) and wild-type (*n* = 3) pigs aged 3 and 10 months. **i** Migration and invasion Transwell assays for pig OS cells transfected with mutant R167H Δ152p53α isoform or a GFP control vector. Left, representative microscopic image (scale bars, 200 μm). Right, quantification of the indicated migrated and invaded cells numbers.

To confirm promoter function five luciferase reporter constructs were generated containing the P1 promoter (−2000 bp to TSS), the putative P2 promoter (intron 4) and three different fragments covering the first intron (Pint_1, Pint_2, Pint_3), see Fig. 2a. Luciferase expression driven by the SV40 promoter was used as positive control.

Luciferase activity was measured after transfection of various porcine primary cells: wild-type bone marrow mesenchymal stem cells (bmMSC) and kidney fibroblasts; fl*TP53*^*R167H/R167H*^ OS cells and healthy osteoblasts; and human HEK293 cells. In comparison to the positive control two of the putative promoter fragments led to moderate (P1, 40–70% and Pint_1, 30–45%) and one to low (P2, 10–25%) luciferase activity (Fig. 2a). These three promoter fragments shared the greatest homology with the corresponding human *TP53* sequence. The in silico and experimental data provide evidence for the presence of internal promoters within intron 1 (Pint) and intron 4 (P2) of the porcine *TP53*, similar to those in human *TP53*.

Expression from the P1 and Pint promoters is blocked by lox-stop-lox cassette in *flTP53*^*R167H*^ homozygous pigs. Additional deletion of the P2 promoter fragment should result in a complete inactivation of porcine *TP53*. Using the Crispr-Cas9 system and two guide RNAs a DNA fragment from 9665 to 10,374 bp of pig *TP53* (NC_010454) was excised in OS cells from *flTP53*^*R167H*^ homozygous pigs (Supplementary Fig. 3a). RT-PCR analysis showed no *TP53* expression in the edited porcine OS cells (Supplementary Fig. 3b), confirming that the P2 promoter is responsible for the Δ152p53α expression.

### The R167H-Δ152p53α isoform is overexpressed in tumours

To assess the RNA and protein expression of Δ152p53α isoform in cancer, we analysed tumours collected from fl*TP53*^*R167H*^ pigs. Quantitative PCR and western blot revealed more than three-fold (*P* < 0.001) higher expression of R167H-Δ152p53α isoform in OS compared to matched healthy bones (Fig. 2b–d). The level of Δ152p53α mRNA expression increased (*P* = 1.17 × 10^–5^) with OS tumour size (Fig. 2e).

The expression level of the Δ152p53α mRNA in healthy bones correlated with the onset of cancer clinical symptoms in homozygous fl*TP53*^*R167H*^ pigs (Fig. 2f). The higher R167H-Δ152p53α was expressed the earlier tumours became evident. In wild-type pigs the level of Δ152p53α expression remained unchanged with age (Fig. 2f).

The above results hinted that overexpression of the mutant R167H-Δ152p53α isoform was essential for tumorigenesis. To assess if this also applied to other tumour types samples from the nephroblastomas and B-cell lymphomas were analysed. As for the OS, an overexpression of Δ152p53α isoform was observed in kidney and spleen tumours (Fig. 2g). Finally, we also tested if the orthologous Δ133/160p53 mRNA was expressed in human OS samples. These too were positive (Supplementary Fig. 4).

Overexpression of the Δ152p53α isoform seemed essential for tumour growth. To confirm this, and to assess if the effect was due to the expression of the wild-type or mutant isoform, a proliferation assay was performed. Pig OS cells transfected with an expression vector carrying the wild-type Δ152p53α or mutant R167H Δ152p53α cDNA sequence under the control of the CAG promoter showed significantly increased proliferation compared to those transfected with control GFP vector (Supplementary Fig. 5). The highest proliferative increase was obtained for the mutant isoform. Next, we tested if expression of the mutant R167H Δ152p53α isoform also increases cell migration and invasion. Compared to the control a significant difference was observed (Fig. 2i).

The MDM2 oncoprotein is a key regulator of p53 expression, which is stabilised by mutant p53 [33]. To determine if the mutant Δ152p53α protein isoform retains this function, western blot analysis was carried out and showed an increased MDM2 protein expression in OS tumour samples (Fig. 2c). Taken together, these data indicate that the mutant Δ152p53α isoform plays a critical role in the malignant transformation of bones, kidney or spleen in fl*TP53*^*R167H*^ pigs.

### Blood exosomal Δ152p53α expression is indicative of tumorigenesis

In order to investigate whether the increased expression of R167H-Δ152p53α mRNA during malignancy can be detected in serum samples and used as a biomarker, exosomes were isolated from *flTP53*^*R167H*^ heterozygous (*n* = 6), homozygous (*n* = 6) and wild-type (*n* = 3) pigs aged 3 and 10 months. At the age of 3 months all animals were disease free while at the age of 10 months the *flTP53*^*R167H*^ homozygous pigs showed first signs of cancer, confirmed later by necropsy. QPCR showed a six-fold higher level (*P* < 0.01) of R167H-Δ152p53α mRNA in exosomes from 10-month old *flTP53*^*R167H*^ homozygous pigs than from the same pigs at the age of 3 months (Fig. 2h). The exosomal Δ152p53 mRNA expression in *flTP53*^*R167H*^ heterozygous and wild-type pigs was low and unchanged over the same time period (Fig. 2h). These data suggest that detection of Δ152p53α isoform mRNA in serum is indicative of tumorigenesis.

### The DNA methylation in the P2 promoter is negatively correlated with the mutant Δ152p53α expression in OS cells and tumours

**Fig. 3:**
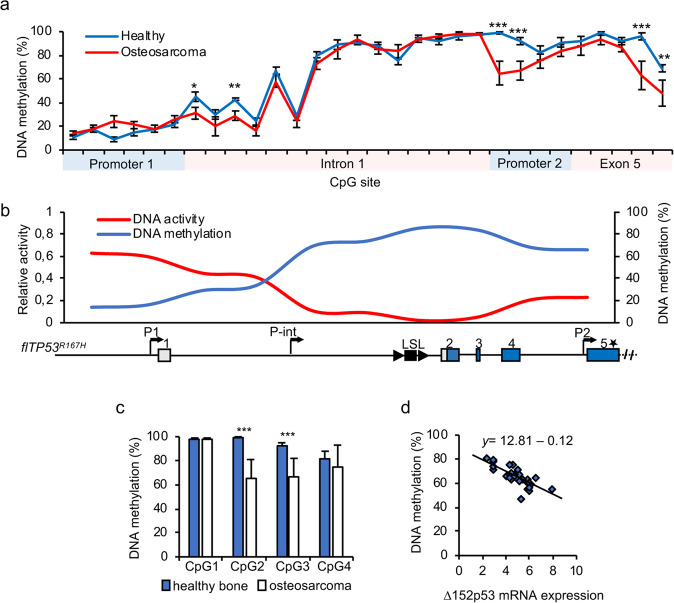
DNA methylation level of porcine *TP53* promoters. **a** DNA methylation analysis of CpGs located in putative promoter regions *in TP53* in osteoblasts and OS cells of *flTP53*^*R167H/R167H*^ pigs. **b** The correlation plot for promoter activity and DNA methylation. The values on the plot are aligned to the *TP53* gene structure. **c** Pyrosequencing analysis of four CpG sites in P2 promoter (P2 fragment) in OS (*n* = 48) and matched healthy bone (*n* = 29) of fl*TP53*^*R167H*^ pigs. Values shown represent mean ± SD. **P < 0.01, ***P < 0.001. **d** Regression analysis between CpG methylation at site 2 in P2 promoter and Δ152p53α mRNA expression in OS (*y* = 12.81–0.12, *P* = 2.9 × 10^–^^7^).

The P2 promoter showed an increased activity in tumours. It has been reported that intragenic *TP53* methylation differs between normal and transformed human colorectal cancer cell lines [34]. To evaluate if epigenetic changes correlate with altered expression levels a comparison of the DNA methylation in healthy osteoblasts and OS cells derived from *flTP53*^*R167H/R167H*^ pigs was carried out. CpG regions in the P1 promoter, intron 1 (fragments Pint_1, Pint_2, Pint_3), P2 promoter and exon 5 were identified. DNA methylation at four or more CpG sites was analysed for each region. Overall, CpG methylation was higher in the gene body than in the P1 promoter (Fig. 3a), which is consistent with *TP53* methylation pattern in human [35]. The highest DNA methylation (>80%) was observed in Pint_2 and Pint_3 fragments that showed no promoter activity. The genomic regions of P1, Pint_1 and P2 promoters showed 18%, 28%, and 64% DNA methylation in healthy osteoblasts, respectively. The DNA methylation at five CpG sites in Pint_1, P2 and exon 5 was significantly lower in OS than in healthy osteoblasts cells (Fig. 3a). The level of DNA methylation in osteoblasts and OS cells inversely correlated with promoter activity (Fig. 3b).

The same analysis was carried out for OS tissue samples. Compared to healthy bone the same five CpG sites as mentioned above showed significantly reduced DNA methylation in OS. The level of DNA methylation was as follows: in Pint_1—CpG3 (41% vs. 22%, healthy bone vs. OS, *P* < 0.01), in the P2 promoter—CpG2 (99% vs. 62%, *P* < 0.0001) and CpG3 (96% vs. 65%, *P* < 0.0001) site, and CpG5 (94% vs. 63%, *P* < 0.01) and CpG6 (67% vs. 52%, *P* < 0.01) site within the Kozak sequence (Fig. 3c and Supplementary Fig. 6e). CpGs in this region have been shown to be differentially methylated for the Δ133/160 isoforms in human [34]. The regression analysis revealed that the decreased methylation at CpG2 and CpG3 sites in the P2 promoter is correlated with higher R167H-Δ152p53α mRNA expression in OS (*P* = 2.9 × 0^−7^; Fig. 3d).

### *TP53* circular RNA enhances proliferation of OS cells

**Fig. 4:**
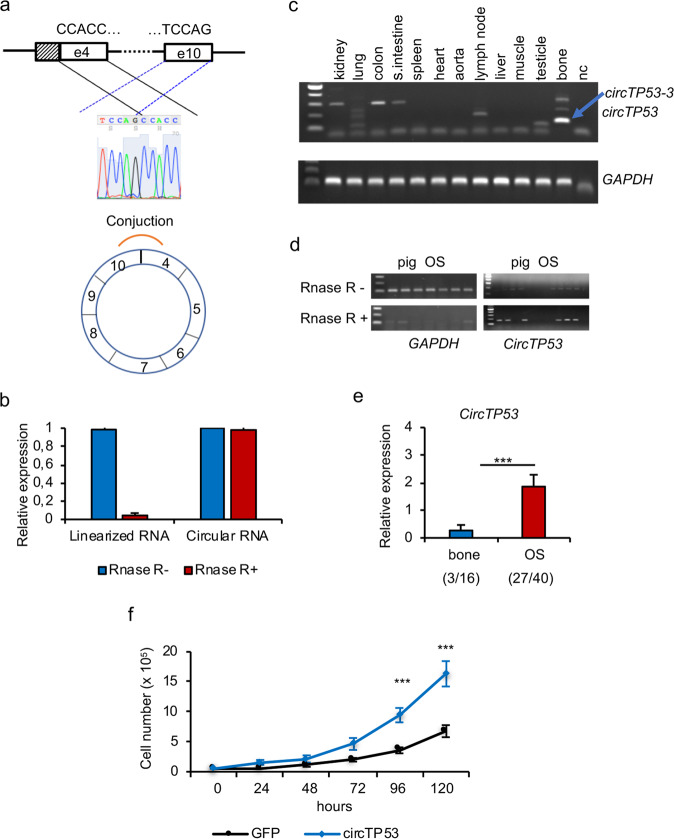
Identification of *circTP53* in *flTP53*^*R167H*^ pigs. **a** Schematic presentation of *circTP53* identification and confirmation by sanger sequencing. **b** Q-RT-PCR results showing the effect of RNAase R digestion. **c** RT-PCR analysis of circTP53 in different tissues in *flTP5*^*R167H/R167H*^ pigs. **d** RT-PCR amplification showing the effect of RNase R digestion on *circTP53* and *GAPDH* amplification. **e** Relative expression of *circTP53* in healthy bones and OS. In parenthesis the number of *circTP53* positive to analysed samples is shown. **f** Proliferation assay in pig fl*TP53*^*R167H/R167H*^ OS cells transfected with *circTP53* overexpression vector, consisting of exon 5 to exon 9 (circ*TP53-3*) under the control of CMV promoter.

There is increasing evidence that circular RNAs (circRNAs) play a role in human cancer [36, 37], but so far no data have been reported showing expression of a circRNA for *TP53* (*circTP53*). To identify *circTP53* in *flTP53*^*R167H/R167H*^, several pairs of divergent primers were designed for RT-PCR analysis resulting in the detection of four different circ*TP53* in fl*TP53*^*R167H/R167H*^ tissues (*circTP53-1 to -4*, Fig. 4a), of which *circTP53-3 was highly expressed in bone*. All were expressed from the P2 promoter and encoded within the Δ152p53α isoform, which was confirmed by Sanger sequencing and RNase R digestion (Fig. 4b). RT-PCR analysis revealed a tissue-specific expression of *circTP53* variants, with their highest expression in bone, kidney and colon (Fig. 4c). No expression of *circTP53* was found in heart and aorta, which is consistent with the lack of Δ152p53α expression in these organs. Compared to healthy bones, the *circTP53* expression was significantly increased (*P* < 0.001) in OS (Fig. 4d, e).

To test, whether the newly detected *circTP53* has any effect on cell proliferation, an overexpression vector, consisting of exon 5 to exon 9 (circ*TP53-3)* and the R167H mutation under the control of CMV promoter, was generated. As shown in Fig. 4f, the proliferation of pig OS cells overexpressing the *circTP53* was significantly increased (*P* < 0.001) compared to cells transfected with a control GFP vector.

### Altered expression of p63 and p73 in tumours from *flTP53*^*R167H*^ pigs

The p53 family includes two other members: p63 and p73, all three genes are structurally similar, and have been implicated in cell regulation and cancer [38, 39]. This raised the question whether the *TP53* mutation also influenced the expression of p63 and p73 in fl*TP53*^*R167H*^ pigs.

#### p63

**Fig. 5:**
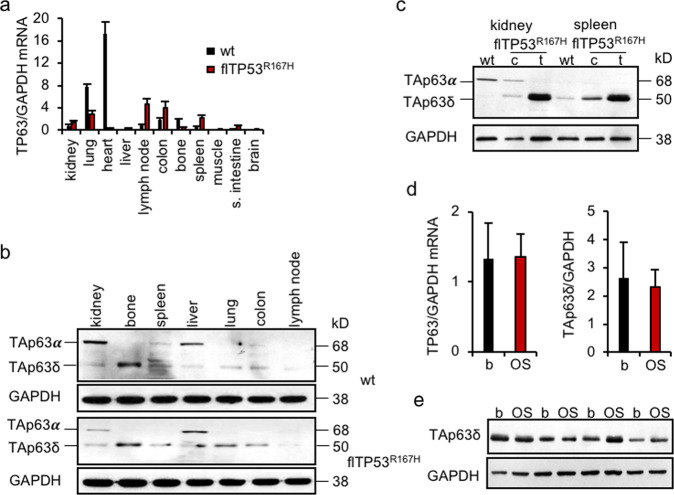
Expression of p63 in *flTP53*^*R167H*^ pigs. **a** Quantitative RT-PCR analysis of different tissues (*n* = 11) from *flTP5*^*R167H/R167H*^ (*n* = 6) and wild-type (*n* = 3) pigs. **b** Representative western blots showing p63 protein expression in different tissues of *flTP5*^*R167H/R167H*^ and wild-type pigs. **c** Western blots showing p63 isoforms expression in healthy and tumour tissues of kidney and spleen of *flTP5*^*R167H/R167H*^ and wild-type pigs. **d** Quantitative PCR results of p63 mRNA expression in OS (*n* = 48) and matched healthy bone samples. **e** Representative western blots showing p63 protein expression in OS and healthy matched bone samples of homozygous *flTP53*^*R167H*^ pigs. Quantitative measurements of protein in OS (*n* = 10) and matched healthy bone samples of homozygous *flTP53*^*R167H*^ pigs.

p63 has two major isoforms, TAp63 and ΔNp63, which have different roles in tumorigenesis. While ΔNp63 isoform promotes, the TAp63 suppresses tumour growth in mice [40]. Different tissues (*n* = 11) from fl*TP53*^*R167H*^ homozygous (*n* = 10) and wild-type (*n* = 3) pigs were analysed by qPCR with primers located in exon 3 and exon 4. The expression of the detected *TP63* mRNA variant was significantly lower in heart, lung, bones and higher in lymph node, colon, spleen in fl*TP53*^*R167H*^ homozygous compared to wild-type pig tissues (Fig. 5a). Western blot analysis revealed tissue-specific expression of two TAp63 isoforms, TAp63α and TAp63δ and no expression of ∆Np63 (Fig. 5b). While the TAp63α isoform was highly expressed in wild-type kidney, the TAp63δ showed an overexpression in wild-type bones, and kidney and spleen tumours (Fig. 5c), and variable expression in OS (Fig. 5d) from fl*TP53*^*R167H*^ pigs, indicating that the *TP53* mutation has a tissue- and tumour-specific effect on the TAp63δ isoform expression.

#### p73

**Fig. 6:**
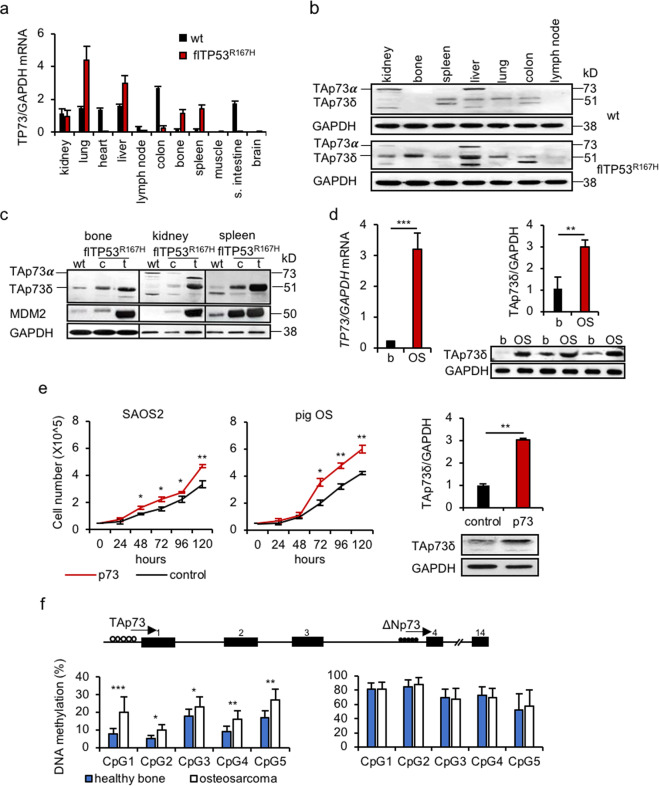
Expression of p73 in *flTP53*^*R167H*^ pigs. **a** Quantitative RT-PCR analysis of p73 mRNA expression in different tissues (*n* = 11) of *flTP5*^*R167H/R167H*^ (*n* = 6) and wild-type (*n* = 3) pigs. **b** Western blots showing the tissue-specific p73 isoforms expression in *flTP5*^*R167H/R167H*^ and wild-type pigs. **c** Western blots showing p73 isoforms and MDM2 proteins expression in bone, kidney and spleen tumours and healthy matched tissues of *flTP5*^*R167H/R167H*^ and wild-type pigs. **d** Quantitative PCR (left) and western blot (right) measurements showing p73 expression in OS (*n* = 48) and matched healthy bone samples. Representative western blots (bottom) showing TAp73δ isoform expression in OS and matched healthy bone samples of homozygous *flTP53*^*R167H*^ pigs. Quantitative measurement of western blots was performed for OS (*n* = 10) and matched healthy bone (*n* = 10) samples of homozygous *flTP53*^*R167H*^ pigs. **e** Proliferation assay in human SAOS2 (left) and pig *flTP53*^*R167H/R167H*^ OS (middle) cells. Western blot (right) showing the upregulation of the TAp73δ isoform in pig OS cells transfected with a full length of *TP73* cDNA vector. The bar plot above shows the quantitative protein measurements (*n* = 3) of western blots. **f** DNA methylation analysis of P1 and P2 promotor regions of *TP73* in OS (*n* = 48) and matched healthy bone samples of *flTP53*^*R167H*^ pigs. Open and filled circles on the *TP73* gene structure indicate unmethylated and methylated DNA regions.

In humans, p73 has two different promoters, which regulate several isoforms: full length TAp73 and N-terminal truncated ∆Np73, which can be distinguished by their transactivation functions [41, 42]. It has been suggested that TAp73 has a tumour suppressor function similar to that of p53, whereas ∆Np73 isoforms would promote cell growth by regulating activities of p53 family members [43]. QPCR analysis revealed a high *TP73* mRNA expression in kidney, lung, liver, colon, spleen, bone, lymph node and no expression in muscle and brain of wild-type animals (Fig. 6a). It was significantly lower in colon and small intestine but higher in lung, bone, spleen of fl*TP53*^*R167H*^ homozygous pigs compared to wild-type (Fig. 6a). Western blot detected the TAp73α isoform in kidney and liver and the TAp73δ isoforms in spleen, liver, lung of fl*TP53*^*R167H*^ homozygous and wild-type pigs (Fig. 6b). Compared to wild-type, an overexpression of TAp73δ isoform was observed in healthy bones (Fig. 6b) and tumours (Fig. 6c, d) from fl*TP53*^*R167H*^ homozygous pigs.

We next determined if the p73 isoforms expression is correlated with DNA methylation in the two promoter regions. A hypermethylation of the ∆Np73 promoter and a hypomethylation of the TAp73 promoter was observed. The DNA methylation at all five CpG sites of TAp73 promoter differed significantly, in particular at CpG1 (21% vs. 9%, *P* < 0.001) between OS and matched healthy bone samples (Fig. 6f), which suggests that the CpG sites may be involved in the regulation of the TAp73 isoform expression.

To further investigate the function of p73 in tumorigenesis, a porcine *TP73* full length cDNA expression vector, containing both the TAp73 and ∆Np73 translation start codons, was transfected into porcine OS and human OS (SAOS2) cells. The *TP73*-transfected cells showed an overexpression of the TAp73δ, no expression of ∆Np73 isoform (Fig. 6e) and significantly (*P* < 0.01) increased growth rate compared to the GFP transfected control cells. These data indicated that the TAp73δ isoform is predominantly translated from the *TP73* cDNA and has an effect on the proliferation of these cells.

In summary, these analyses showed for the first time the expression of p63 and p73 isoforms in porcine tissues and the upregulation of the two isoforms (TAp63 and TAp73) presumed to have tumour suppressor function in porcine tumours. However, overexpression of TAp73δ did not lead to a reduction but to an increase in cell proliferation, which may confirm that different C-terminal variants may lack growth suppressing function.

## Discussion

It is becoming evident that p53 isoforms, including those derived from the internal P2 promoter, have implications in human cancers [8]. Further elucidation of their roles, interactions, regulation and patterns of expression could open novel approaches for the prognosis and treatment of cancer. But the study of p53 isoforms and its involvement in tumorigenesis had been hampered by the fact that the main experimental mammal, the mouse, lacks internal P2 promoter activity [8]. This motivated the generation of mice that ubiquitously express a Δ133p53-like protein (Δ122p53) through deletion of exon 3 and 4 [44, 45]. Homozygous Δ122p53 mice show an enhanced proinflammatory phenotype and are prone to develop B-cell tumours [46] with low incidence of OS (17%) [45]. Although this mouse model does not replicate the situation in human, its strongly supports the notion that p53 isoforms play a role in cancer.

The study presented here shows that the porcine *TP53*—unlike the mouse—has two internal promoters, Pint in intron 1 and P2 in intron 4. In humans the expression from the P2 promoter results in two isoforms, Δ160p53 and Δ133p53. The porcine Δ152p53 protein isoform is equivalent to human Δ160p53. The pig lacks the transcription initiation site, which in humans enables translation of the Δ133p53 protein isoform. Three N-terminal variants have been observed in humans (p53α,β,χ). The porcine mRNA isolated by RT-PCR represents the Δ152p53α mRNA. However, western blot analysis indicated that N-terminal variants are present in the pig.

To our best knowledge this is the first report showing an association between P2 promoter activity and epigenetic modifications in normal and tumour tissue. This is an important finding as epigenetic modulation has been suggested as a means of restoring wild-type p53 function, or inactivating mutant p53 activity in human cancer [47].

Comparison with human data shows that the porcine Δ152p53α mRNA is expressed in a similar tissue-specific manner, including high expression, e.g., in bone and lack of expression in heart tissue [7]. Our study strongly suggests that the predominance of OS followed by nephroblastomas and B-cell lymphomas in pigs carrying the *floxed TP53*^*R167H*^ allele is related to the higher level of Δ152p53α expression in these organs. This is further supported by the finding that pigs with early onset of OS have also higher expression of the Δ152p53α isoform in healthy bone tissue.

Expression of Δ152p53α isoform increases during tumorigenesis, which is consistent with the expression of p53 isoforms in human cancers [48]. The overexpression of Δ133/Δ160p53 variants and their potential oncogenic function have been reported in lung, colon, breast and ovarian cancers and in melanoma [49–52]. In the pig, an overexpression of the wild-type and mutant R167HΔ152p53α isoform enhanced cell proliferation, finding consistent with data for mutant Δ160p53 isoform in human cancer cells [53]. It was more pronounced for mutant isoform, which would imply that the tissue-specific expression of the mutant Δ152p53α isoform drives tumorigenesis in our pig model and is an indicative blood biomarker.

*TP53*^*R167H*^ Yucatan minipig model generated by Sieren et al. [54] develops a similar tumour spectrum, but the role of the Δ152p53 in this model is unknown. It is questionable if the P2 promoter is functional in this model due to the insertion of a selectable marker gene in intron 4.

It has been suggested that one of the key mechanisms for p53 gain of function mutations is its interaction with p63/p73 [55, 56], and that the ratio of TA/ΔNp63/p73 isoforms determines their effect on tumorigenesis [57, 58]. We observed an upregulation of the TAp63δ and TAp73δ isoforms in all studied tumours, and proved that experimental overexpression of the TAp73δ isoform increased proliferation of human and porcine OS cells. Further research is required to prove a direct interaction between the specific isoforms.

We present the first description of *circTP53* RNAs expressed from the P2 promoter. As with the parental Δ152p53α isoform it is upregulated in tumours such as OS. Importantly, the functional study demonstrated that high *circTP53* expression increases cellular proliferation of OS cells. Similar mechanisms for gene overexpression and upregulation of circRNA was described for other genes, e.g., androgen receptor in prostate cancer [59]. The mechanism how *circTP53* affects cell proliferation still needs to be elucidated.

## Conclusion

The biology of p53 has been studied extensively for 4 decades and still novel insights are being gained. A cross-species comparison might help to understand the tissue-specific regulation of *TP53*. Our study has highlighted the value of using the pig model. We show that the Δ152p53 isoform, its circular counterpart and the p53 family members, TAp63δ and TAp73δ, likely play a role in the malignant transformation of bone and other tumours. Considering the similarities of *TP53* regulation between pig and human, the observations presented here provide useful information for further studies on the regulation of p53 in humans.

## Material and methods

### Animals

Three wild-type, ten (8 males and 2 females) *flTP53*^*LSLR167H/R167H*^ and 24 (13 males and 11 females) *flTP53*^*LSLR167H/+*^ pigs aged 7–32 months were produced by normal breeding and raised in our animal husbandry facilities with food and water provided ad libitum.

### Necropsy examination and tumour analysis

In total, 51 pigs were euthanised and examined by complete necropsy (without randomisation). Three bone samples from wild-type and 48 OS samples with matched healthy bones from hetero- and homozygous fl*TP53*^*LSLR167H*^ pigs were collected and all samples were included in the analysis. The study was non-blinded.

### Porcine primary cells

Porcine bmMSC and kidney fibroblasts were derived in-house and cultured by standard procedures [20]. All cell cultures were routinely tested for mycoplasma.

### Primers

All primers used are shown in Supplementary Table S1.

For quantitative PCR, 5’ and 3’RACE analysis, RNase R digestion, pyrosequencing, functional assays, western blot and sgRNA constructs details see Supplementary text. Unprocessed western blots are shown in Supplementary Fig. 7.

## Supplementary information

Supporting Information

## Data Availability

All supporting data are included in the Supplementary file.
