# Supplementary material for: Porcine model elucidates function of p53 isoform in carcinogenesis and reveals novel circTP53 RNA
Source: Oncogene. 2021 Feb 18;40(10):1896–908. doi: 10.1038/s41388-021-01686-9 (PMC7946636; doi:10.1038/s41388-021-01686-9)
Supplement: Supplementary file 1 — Supporting Information [file 41388_2021_1686_MOESM1_ESM.docx]

Supplementary information

**Porcine model elucidates function of p53 isoform in carcinogenesis and reveals novel *circTP53* RNA**

Guanglin Niu^1^, Isabel Hellmuth^1^, Tatiana Flisikowska^1^, Hubert Pausch^2^, Beate Rieblinger^1^, Alexander Carrapeiro^1^, Benjamin Schade^3^, Brigitte Böhm^3^, Eva Kappe^3^, Konrad Fischer^1^, Bernhard Klinger^1^, Katja Steiger^4^, Reiner Burgkart^5^, Jean-Christophe Bourdon^6^, Dieter Saur^7,^ Alexander Kind^1^, Angelika Schnieke^1^, Krzysztof Flisikowski^1^

^1^ Chair of Livestock Biotechnology, Technische Universität München, Germany

^2^ Animal Genomics, ETH Zurich, Switzerland

^3^ Bavarian Animal Health Service, Department of Pathology, Poing, Germany

^4^ Institute of Pathology, School of Medicine, Technische Universität München, Germany

^5^ Klinik und Poliklinik für Orthopädie und Sportorthopädie, Klinikum rechts der Isar, Technische Universität München, Germany

^6^ Jacqui Wood Cancer Centre, School of Medicine, University of Dundee, United Kingdom

^7^ Department of Internal Medicine II, Klinikum rechts der Isar, Technische Universität München, Germany

**Supplemental material and methods**

**Necropsy examination and tumour analysis**

Three bone samples from wild type and 48 OS samples with matched healthy bones from hetero- and homozygous fl*TP53^LSLR167H^* pigs analysed. The size criteria for bone tumours were according to the AJCC Cancer Staging Manual. For histopathology analysis, organ specimens were fixed in 4% buffered formaldehyde, embedded in paraffin, sectioned (4 μm) and stained with haematoxilin and eosin (H&E). Bone specimens were first decalcified in Ossa Fixona (Waldeck GmbH, Germany). For cryosections, normal and tumour samples were preserved in 2-methylbutane (OCT). For molecular analyses they were snap frozen and stored at -80 °C.

**Exosomes isolation from blood**

40 ml of EDTA blood was collected from *flTP53^R167H^* heterozygous (n= 6), homozygous (n= 6) and wild type (n= 3) pigs aged 3 and 10 months. For the isolation and purification of exosomes the ExoEasy Maxi Kit (Qiagen) was used according to manufacturer’s protocol. The exosomal RNA was isolated using the RNeasy Midi Kit (Qiagen).

**Porcine primary cells**

Porcine bone marrow MSCs (bmMSC) and kidney fibroblasts (KDNF) were derived in-house and cultured by standard procedures. All cell cultures were routinely tested for mycoplasma. To minimise phenotype changes, cells were kept as frozen stocks and cultured for 4 weeks maximum.

**Porcine osteosarcoma cell culture**

Cells were derived from a minced piece of tumour digested in DMEM supplemented with 200U/ml collagenase type IV (Worthington, USA) at 37 °C for 24 h and centrifuged at 100 r.c.f. for 5 min. Cells were resuspended in standard porcine MSC medium (20) and cultured at 37°C, 5% CO_2_ .

**Reverse transcription PCR**

100 ng of total RNA was used for cDNA synthesis using the Superscript IV (Thermo Fisher) according to manufacturer’s protocol. RT-PCR was carried out using the PyroMark PCR mix (Qiagen) and using primers that specifically detect the alternative smaller p53 RNA species. The RT-PCR products were verified as p53 RNA by sequencing.

**Quantitative real-time RT-PCR**

QPCR was carried out using Kapa SYBR @ Fast Mix (Kapa Biosystems Pty, South Africa) and run on ABI 7500 PCR System (Applied Biosystems, USA) with default thermal cycling parameters. Reactions were performed in 10 μl volume. Samples were assayed in triplicate and relative expression was normalised to *GAPDH* expression. Fold-differences were calculated by the _ΔΔ_CT method and statistically compared using Students t-test. The variance between the groups was similar.

**RNase R digestion**

RNase R treatment was carried out for 20min at 37°C using 2U RNase R (Epicenter) per 1μg of RNA. Treated RNA was directly reverse transcribed using Superscript IV (Thermo Fisher) with a random decamer primer according to the manufacturer’s instructions.

**5’ and 3’ Rapid amplification of cDNA ends (RACE)**

### 1 µg of total RNA from OS, bone and kidney samples was used for 5’ and 3’ RACE reactions with the FirstChoice RLM-RACE kit (Ambion) according to manufacturer’s protocol. Modified RNA was reverse transcribed using SuperScript IV (Thermo Fisher). The resulting cDNA was used for nested PCR.

**Pyrosequencing**

Pyrosequencing assays were designed using PyroMark Assay Design 2.0 software (Qiagen). 500 ng genomic DNA was bisulphite-converted with the EZ DNA Methylation-Direct kit (Zymo Research, Irvine, USA) according to the manufacturer’s instructions. PCR samples were amplified using PyroMark PCR kit (Qiagen). PCR products were sequenced using PyroMark Q48 Advanced CpG reagents on a PyroMark Q48 Autoprep instrument (Qiagen). For assay optimisation, methylated (100%), non-methylated (0%) and a scale of control samples with the following DNA methylation: 25%, 50% and 75% were used. The methylated control was prepared using the CpG methyltransferase enzyme (M.SssI; Thermo Scientific). The non-methylated control was prepared using REPLI-g Mini kit (Qiagen).

**Luciferase reporter assay**

Porcine bone-marrow mesenchymal stem cells and kidney fibroblasts were cultured in DMEM supplemented with 20% FCS, 1% sodium pyruvate, 1% NEAA and 1% glutamine. Porcine osteoblasts and OS cells were cultured in DMEM/F12 supplemented with 10% FCS and 1% glutamine. HEK293 cells were cultured in DMEM supplemented with 10% FCS. All cells were plated into 24-well plates and then transfected with P1, Pint_1, Pint_2, Pint_3 and P2 psiCHECK2 plasmids (0.5 µg per well) using Lipofectamine 2000 transfection reagent (Thermo Fischer). An empty psiCHECK2 vector and with the SV40 promoter were used as negative and positive controls. Firefly and *Renilla* luciferase activities were measured using a *Firefly* & *Renilla* Luciferase Single Tube Assay kit (Biotium) on FLUOstar Omega (BMG Labtech). All assays were performed in triplicate.

**Porcine Δ152p53^WT^ and Δ152p53^R167H^ mRNA expression vectors and stable transfection**

Both expression vectors comprised of the CAG promoter directing expression of *Δ152p53^WT^* or *Δ152p53^R167H^* mRNAs linked to bovine growth hormone polyA. These together with hygromycin selectable marker vector were then used for co-transfection of fl*TP53^167H/167H^* OS and wild type kidney fibroblasts porcine cells.

**Proliferation assay**

Porcine OS cells were transfected with *Δ152p53^WT^*, *Δ152p53^R167H^*, and *circTP53* overexpression vectors by electroporation using the EMC830 electroporation system (BTX). GFP vector was used as a control. The transfected cells were selected by using 200ng/μl of hygromycin. After selection, 5 x 10^5^ cells were plated on 6 well- plates (3 times for each assay). The cells were counted after 24h, 48h, 72h, 96h and 120h of incubation using an automated cell counter (Invitrogen).

**Migration and invasion assay**

24 hours before plating, cells were washed and cultured in FCS free medium. 1 x 10^5^ cells (GFP control and mutant R167HΔ152p53α) were plated on 24-well 8.0 μm transwell inserts (Corning Inc.) directly for cell migration assay, or after coating with 10% Matrigel for the cell invasion assay. Medium with FCS was added at the bottom of each transwell. Cells were incubated for 24 hours, fixed with methanol, stained with Crystal Violet, then washed six times with water, air dried overnight, and the cell number per field was determined. Each experiment was carried out in triplicate.

**Western blot analysis**

Protein was isolated using NP40 buffer, 40 mg of protein was separated on a 12% SDS PAGE gel, transferred to PVDF membrane and processed using the iBind Western System (Thermo Scientific) according to the manufacturer protocol. Pig Δ152p53 isoform was detected using sheep Sapu antibody (diluted 1:1000) and horseradish peroxidase (HRP) labelled anti-sheep s36-62DD (diluted 1:2000). Pig p63, p73 and MDM2 were detected using rabbit anti-p63 monoclonal antibody ab124762 (diluted 1:1000), rabbit anti-p73 polyclonal antibody PA5-80175 (diluted 1:1000), rabbit anti-MDM2 polyclonal antibody ab260074 (diluted 1:1000) and horseradish peroxidase (HRP) labelled anti-rabbit sc-2004 (diluted 1:2000), respectively. GAPDH was detected using mouse monoclonal anti-GAPDH #G8795 (diluted 1:3000) and rabbit anti-mouse IgG H&L (HRP) ab6728 (diluted 1:5000). ECL Plus kit was used for the detection.

**Generation of sgRNA constructs**

SgRNA constructs targeting *TP53* P2 promoter were generated by cloning the respective gRNA oligonucleotides (gRNA_P2_1F:5’-GTAAGGACTGGGGCGCGGCA- 3’; gRNA_P2_1R: 5’-TGCCGCGCCCCAGTCCTTAC- 3’; gRNA_P2_2F: 5’-GCGTCTGTTCATTTGACTGC- 3’; gRNA_P2_2R: 5’-GCAGTCAAATGAACAGACGC- 3’) into  pX330-U6-Chimeric_BB-CBh-SpCas9 vector which digested with BbsI from Feng Zhang (Addgene plasmid # 42230; [http:/n2t.net/addgene:4223O;RRID:Addgene_42230](http://n2t.net/addgene:4223O;RRID:Addgen_42230)). Both sgRNA constructs were cotransfected into pig OS cells.

**Table S1**. List of primers used in the study.

| Primer Name | Sequence |
| --- | --- |
| TP53_1F | GGTTCCTGCAATCTGGAACA |
| TP53_1R | ATTCCCTTCCACCCGGATGA |
| TP53_2F | TCACCGGGTGGAAGGGAAT |
| TP53_2R | GCTGTTACACATGAAGTTGT |
| TP53_5F | TGCAGCTGTGGGTCAGCTCG |
| TP53_4R | AACACGCACCTCAAAGCTGT |
| TP53_5R | CGCCATCCAGTGGCTTCTTC |
| TP53_9F | TCCTGCAGTACTCCCCTGCC |
| TP53_6R | GAAGCTAGGAGAGCGTGTC |
| TP53_8R | TCGGAACATCTCGAAGCGTT |
| TP53_9R | CAGGTCCTTCTCTCTTGAAC |
| TP53_11F | AAAATTTCCTCAAGAAGGGC |
| TP53_21F | GTTCAAGAGAGAAGGACCTG |
| TP53_15F | CTCCATCCTCCCTTTCCTGC |
| TP53_16F | TCCTGCATGGGGGGCATGAA |
| TP53_15R | ACTGAGTAAGAGCAGGAAAC |
| TP53_17F | TGACTGTACCACCATCCACT |
| TP53_18F | ACAGCTTTGAGGTGCGTGTT |
| TP53_19F | TTTCCTGCAGTACTCCCCTG |
| TP53_20F | CCTT TCCTGCAGTA CTCCCCT |
| TP53_11R | TTGGCCCTTCTTGAGGAAAT |
| TP53_14F | AGCCCCCTCTGAGTCAGGAG |
| Tp53_10F | ATCCTCCCTTTCCTGCAGTA |
| Tp53_22F | GAAGAAGCCACTGGATGGCG |
| Tp53_23F | A ACGCTTCGAG TGTTCCGA |
| Tp53_24F | ACAACTTCATGTGTAACAGC |
| Tp53_16R | GAGGAAAGGTGAGAAAAGAG |
| TP53_17R | CAGGAGGTGGCTGGTGTG |
| TP53_18R | TAGACGGAAATCATAGCTGC |
| TP53_19R | CGAGCTGACCCACAGCTGCA |
| Tp53_25F | GCAGCTATGATTTCCGTCTA |
| TP53_27F | ACTGCCCACCAGCACCAGCT |
| TP53_20R | CTATAGTCAGAGCTGCGCTC |
| TP53_MR5 | TCACACAACAACCCCAAATCCTTAA |
| TP53_MF5 | TTGTTTTGGTTTGTTTAGGAAATTTTAAT |
| TP53_MS5 | ATAAGAAATTAATAAATTAGG |
| TP53_MF6 | GTTTGGTTTGAAGGAAGGTAGTT |
| TP53_MR6 | ATTCCCTTCCACCCAAATAA |
| TP53_MS6 | TGTAGTTGTGGGTTAG |
| TP53_P21_1F | AGAGGAACTTGGTTAGGTACTTTAGCCACCGCTTTTGGGA |
| TP53_P21_1R | GCTTTTTGCAAAAGCCTAGGATAGGACGCGAAACCTCGTG |
| TP53_P22_5F | AGAGGAACTTGGTTAGGTACAGTGCAGAGTTGGAGGTCTTA |
| TP53_P22_2R | GCTTTTTGCAAAAGCCTAGGACTGCAGTGGTTTAGGGAAGT |
| TP53_P23_1F | AGAGGAACTTGGTTAGGTACCCTGCCATCAGGAACAACGA |
| TP53_P23_1R | GCTTTTTGCAAAAGCCTAGGAGTGCTCCTCGTGCTTACAC |
| TP53_P24_1F | AGAGGAACTTGGTTAGGTACGGAGCTTGCCCTTCAGTGAT |
| TP53_P24_1R | GCTTTTTGCAAAAGCCTAGGCGAGCTGACCCACAGCTGCA |
| TP53_P11_2F | AGAGGAACTTGGTTAGGTACACCTGTG GCCTATGCAG GTT |
| TP53_P11_1R | GCTTTTTGCAAAAGCCTAGG GGCCCTGGACTTTTGAGGAG |
| TP53_MF1 | AGTTAAGAATTGGTTGGATGAAAATTTAGA |
| TP53_MR1 | AAACCAATCCCTCAAAACCACTAACC |
| TP53_S1 | GGTTGGATGAAAATTTAGATG |
| TP53_MF2 | GTTTTTGTATGGGGGGTATGA |
| TP53_MR2 | AAAAACCTCAACTCCAACTAATC |
| TP53_MS2 | GAAGATGTTAGGTAGGG |
| TP53_pF1 | AGGGAGTCCATCTAAAAGTG |
| TP53_pR1 | ACCTCTTCAGAGTAGGTGCT |
| TP53_pF2 | TAGCACAGATGTGGGCAGAA |
| TP53_pR2 | TAGCTCCCAATGATGACAGG |
| TP53_pF3 | AGAGCCTCACCACGGGTGAG |
| TP53_pR3 | CCAGGCACTGTCCCTACGAA |
| TP53_pF4 | TTCGTAGGGACAGTGCCTGG |
| TP53_pR4 | TTCCACCCGGATGAGATGCT |
| GAPDH_1F | TTCACGACCATGGAGAAGGC |
| GAPDH_1R | GGTTCACGCCCATCACAAAC |
| TP73_X4_1F | GGGCCAGGAT TCCCGGAGCT |
| TP73_X4_1R | CGACGGCGGAAGATCAAAAT |
| TP73_X4_2F | TCCAC CTTCGACACC ATGTC |
| TP73_X4_2R | TGCTCCGCCTTCTTGTAGAT |
| TP73_X4_3F | GGCGGCCCATCCTTATCATC |
| TP73_X4_3R | CTGCTCGCGGTAGTGATCTT |
| TP73_X4_4R | CGCCTCTTCTTCACGTTGGT |
| TP73_X4_4F | GCCAGGTGTGCGAAGATGTC |
| TP73_X4_5R | TCAGTTGGCCTCGCTCTCTG |
| TP73_X4_6R | TTTGCTCATGGGTGAGAGGA |
| TP73_X4_7R | GTTGTTGAGTATCCCTGCGC |
| TP73_X4_8R | CCCCGAGGTCCTCTATCGTT |
| TP73_X4_9R | CTCGATGCAGTTTGGACACC |
| TP73_X4_10R | CAGGCCCTCCTTGATCTTCG |
| TP73_X4_5F | GGAGAACTTTGAGATCCTCA |
| TP73_X4_6F | TCCTCTCACCCATGAGCAAA |
| TP73_cmv_4F | CGACTCACTATAGGGGCCGGCCGGCCAGGTGTGCGAAGATG |
| TP73_cmv_5R | TGTCTGCTCGAAGCGGCCGGCCTCAGTTGGCCTCGCTCTC |
| TP73_X5_1F | CTTCCACCGCTCCAATGTCA |
| TP73_X1_1F | ATTCAAACAGAAACTGCCGGG |
| TP73_X6_1F | TTGTGTCCTGAGAGGGACAGT |
| TP73_X6_1R | GCCCTACTGCAAAATGGCG |
| TP73_X8_1F | CCCACATCTTCCAGAGCGTC |
| TP73_X8_1R | TCCATGGTACTGCTCAGCAA |
| TP73_X7_1F | TCCTTTACTCTGCTGGGGGA |
| P73_MF1 | GTGGATATAGTAGTAGGGT |
| P73_MR1 | ACCAAAATTACCACTCAAAACTCC |
| P73_MS1 | GATATAGTAGTAGGGTTT |
| P73_prom_1F | CTTGCTCGTACCCCTAAGCC |
| P73_prom_1R | GTTCGTGCATGATCTCGTCG |
| TP73_X4_P1.5kb_1F | TGGGTTTGAGTTTTTTGGTGG |
| TP73_X4_P1.5kb_1R | CACCTCTTTACTACTCACCTACT |
| P73_x7_pF1 | GTTTTGAAGAGTTAAAGGTG |
| P73_x7_pR1 | CCAACAATCCATTCTTTAATAC |
| P73_x8_pF1 | TAGAAGGGTGGTTTTAAAGT |
| P73_x8_pR1 | ACCACCACCCCCCTTTAA |
| P73_x7_MS | TTTTTTATGTTTTAGTTAA |
| P73_x8_MS | AGGGTGGTTTTAAAGTT |
| TP73_X4_P1.5kb_MS | TTTTTTGGTGGTTTTTT |
| P73_MS2 | TTAGTTTTAGGTAG |
| P73_prom_2F | CACAGCAGTAGGGCTCCGGC |
| P73_prom_3F | CCAGTCCCAGGCAGGCGGCC |
| P73_prom_2R | TGAAGCGAGTGCGGCTGGGC |
| TP63_X2_1F | GACCCTTACATCCAGCGGTT |
| TP63_X2_1R | CCTGCATGCGAATACAGTCC |
| TP63_X2_2R | GGCGTGGTCTGTGTTGTAGG |
| TP63_X2_2F | CAGTACCTTCCTCAACACACGA |
| TP63_X2_3R | AGGAAGACTGAGACTGCATCG |
| TP63_X5_1F | AGGACGTTCTTTGAACTGGCA |
| TP63_X6_1F | TGGAGCCAGAAGAGAGGACA |
| TP63_X9_1R | GGCGTCAGATTGTTTCGGGG |

Pos.

Human 160 MAIYKQSQHMTEVVRRCPHHERCSD-SDGLAPPQHLIRVEGNLRVEYLDDRNTFRHSVVV

MAIYK+S++MTEVVRRCPHHER SD SDGLAPPQHLIRVEGNLR EYLDDRNTFRHSVVV

Pig 152 MAIYKKSEYMTEVVRRCPHHERSSDYSDGLAPPQHLIRVEGNLRAEYLDDRNTFRHSVVV

Human 219 PYEPPEVGSDCTTIHYNYMCNSSCMGGMNRRPILTIITLEDSSGNLLGRNSFEVRVCACP

PYEPPEVGSDCTTIHYN+MCNSSCMGGMNRRPILTIITLED+SGNLLGRNSFEVRVCACP

Pig 212 PYEPPEVGSDCTTIHYNFMCNSSCMGGMNRRPILTIITLEDASGNLLGRNSFEVRVCACP

Human 279 GRDRRTEEENLRKKGEPHHELPPGSTKRALPNNTSSSPQPKKKPLDGEYFTLQIRGRERF

GRDRRTEEEN KKG+ E PPGSTKRALP +TSSSP KKKPLDGEYFTLQIRGRERF

Pig 272 GRDRRTEEENFLKKGQSCPEPPPGSTKRALPTSTSSSPVQKKKPLDGEYFTLQIRGRERF

Human 339 EMFRELNEALELKDAQAGKEPGGSRAHSSHLKSKKGQSTSRHKKLMFKTEGPDSD 393

EMFRELN+ALELKDAQ +E G +RAHSSHLKSKKGQS SRHKK MFK EGPDSD

Pig 332 EMFRELNDALELKDAQTARESGENRAHSSHLKSKKGQSPSRHKKPMFKREGPDSD 386

**Figure S1.** Protein sequence alignment of human Δ160p53α and pig Δ152p53α isoform. Dissimilarities between the analysed sequences are indicated as (+) and an empty space.


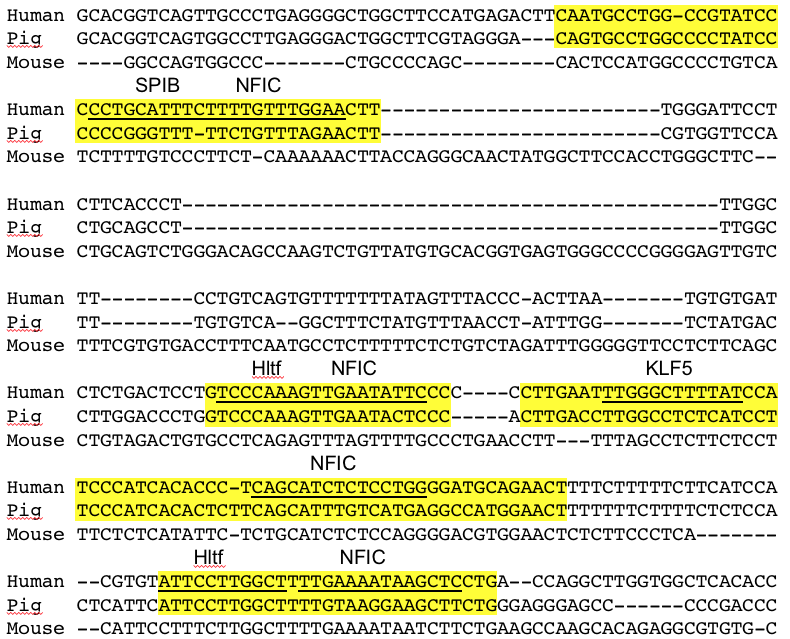


**Figure S2**. Cross-species alignment of *TP53* intron 4 sequence, at the location of the human P2 promoter. Regions of greatest similarity between human and pig are highlighted in yellow. Predicted transcription factor binding sites are underlined. No binding sites for the listed transcription factors were identified in the mouse intron 4.

a


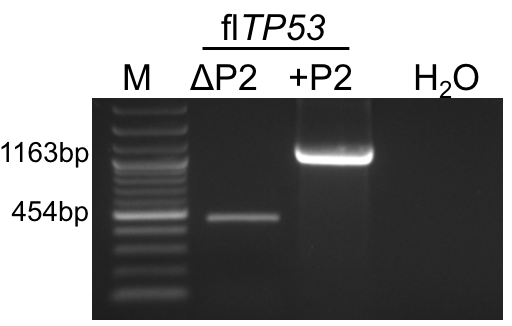


b


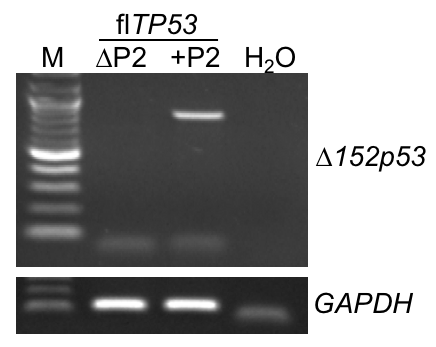


**Figure S3.** Silencing of Δ152p53α expression in pig OS cells. (**a**) PCR showing the CRISPR/Cas9 guided deletion of the *TP53* P2 promoter (9665 to 10374bp on the NC_010454) in *flTP53^R167H^* pig OS cells. (**b**) RT-PCR showing the lack of Δ152p53α expression in the edited pig OS cells. For the RT-PCR, primers specific for the Δ152p53 mRNA expression were used. M - marker, ΔP2 - RT-PCR result in osteosarcoma cells with deleted TP53 P2 promoter, P2 – RT-PCR (807bp) fragment of *TP53* in unedited *flTP53^R167H^* pig osteosarcoma cells, nc – negative control.


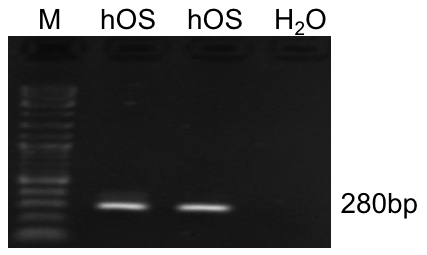


**Figure S4**. RT-PCR analysis of Δ133/160p53 mRNA expression in human OS samples.

*

**

**Figure S5**. Functional analysis of Δ152p53α isoform. Proliferation assay in pig OS cells transfected with an expression vector carrying the wild type Δ152p53α or mutant R167H Δ152p53α cDNA sequence under the control of the CAG promoter. The GFP vector was used as control.

a

b

*

c

d

e

**

**

**Figure S6**. DNA methylation analysis of selected genomic *TP53* regions in OS (n= 48) and matched healthy bone samples of *flTP53^R167H/R167H^* pigs. The analysed fragments included CpG sites in promoter 1 **(a),** fragment Pint_1 **(b),** Pint_2 **(c)**, Pint_ 3 **(d)** in intron 1, and exon 5 **(e)**. Values represent mean ± standard deviation. * P < 0.05, ** P < 0.01.


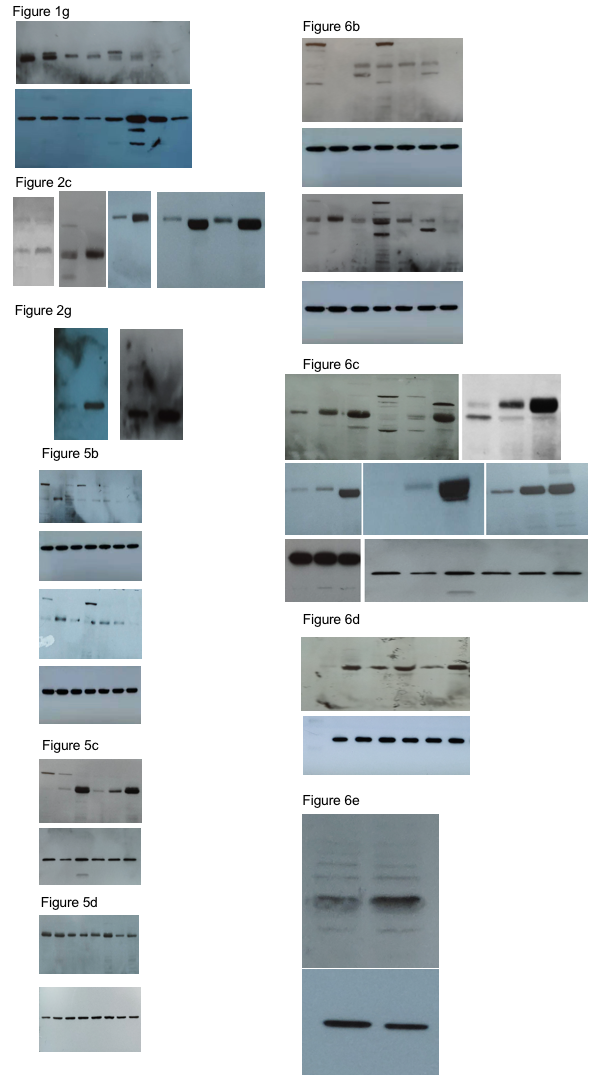


**Figure S7**. Unprocessed western blots.
